# How Social Experiences Affect Interpretation Bias Among Individuals With Non-clinical Depression: The Role of Ostracism

**DOI:** 10.3389/fpsyt.2022.819143

**Published:** 2022-02-07

**Authors:** Avigail Bar-Sella, Thalia Richter, Sigal Zilcha-Mano, Hadas Okon-Singer

**Affiliations:** ^1^Department of Psychology, School of Psychological Sciences, University of Haifa, Haifa, Israel; ^2^The Integrated Brain and Behavior Research Center (IBBR), University of Haifa, Haifa, Israel

**Keywords:** interpretation bias, ostracism, depression, Cyberball task, social cognition, cognitive bias

## Abstract

**Background:**

Extensive knowledge and research indicate that interpretation bias is very common among individuals with sub-clinical and clinical levels of depression. Nevertheless, little is known about the role of social experiences in enhancing interpretation bias. Given the major relevance of social experiences in the context of depression, the present study investigated the role of potential interactions between social experiences and levels of depression symptoms in the interpretation of ambiguous information.

**Method:**

Seventy participants underwent a laboratory controlled manipulation either of social ostracism or of overinclusion. Participants completed a computerized task that measured both direct and indirect interpretation bias and reported their level of depression symptoms.

**Results:**

The findings show that ostracism enhanced interpretation bias when symptom levels were higher, while overinclusion did not. This interaction effect between social ostracism and symptom level was found both for direct and for indirect interpretation bias.

**Conclusion:**

Whereas previous research showed the existence of interpretation bias among people with symptoms of depression, the present study expands previous knowledge by shedding light on the conditions under which interpretation bias emerges, suggesting that ostracism enhances negative interpretation of ambiguous information when levels of depression symptoms are higher.

## Introduction

Our perceptions of reality can be highly subjective and can change as our experiences change. When these experiences are negative, we may interpret information in a negative manner, regardless of its objective manifestation. Beck and Clark (1) defined this tendency as *interpretation bias*: the tendency to selectively interpret ambiguous information in a negative manner. Interpretation bias has been widely studied in sub-clinical and clinical populations and found to be highly related to mental disorders such as depression (2, 3). Accumulating evidence shows that individuals with symptoms of depression, even sub-clinical populations, tend to systematically interpret ambiguous information in a negative manner (2, 4). This association has been replicated in a large number of studies and was found to have a medium effect size, as revealed in a recent meta-analysis (4). Yet, while the existence of interpretation bias among individuals with symptoms of depression is well-established, little is known about the conditions under which this maladaptive cognitive performance may be enhanced.

Theoretical knowledge suggests that external events such as social experiences may enhance interpretation bias among individuals with depression symptoms (5). According to these theories, individuals who exhibit symptoms of depression process information through latent negative cognitive schemas (5). Cognitive schemas, which are defined as internally stored representations of stimuli, ideas, or experiences (6), constitute the central structure in information processing, through which the individual unconsciously grants meaning to new information. When depressed individuals undergo adverse experiences, their negative cognitive schemas are activated and guide their interpretation of the situation, consequently reinforcing negatively biased forms of interpretations (i.e., “She doesn't wave back at me because she doesn't like me, I am so faulty, why should she like me”). One negative experience that may affect interpretation among individuals with depression symptoms is social ostracism (7). Social ostracism is considered aversive and stressful since it interferes with the individual's sense of belongingness, a major motivation defined as a universal human need (7, 8). Moreover, a recent meta-analysis revealed that ostracism can have an aversive and stressful impact on various psychological conditions (interpersonal, e.g., aggressive behavior, and intrapersonal, e.g., self-esteem). This impact has a large effect size (9).

Beyond the extensive effect of ostracism in the general population (9), studies suggest that ostracism may be especially aversive for individuals who exhibit symptoms of depression [e.g., (10)]. These individuals have been found to be highly sensitive to external cues of rejection (10) and to exhibit extensive concern about being rejected [e.g., (11)]. Corresponding with Beck's theory of cognitive schemas (5), a recent study suggests that sensitivity to ostracism may be partially explained by the interaction of external cues with schema-congruent information processing [i.e., a friend who doesn't wave back reinforces the person's internal belief about being faulty and unlovable; (12)]. This information is instrumental in demonstrating the sensitivity of individuals with symptoms of depression to ostracism. Yet whether the interaction between actual cases of ostracism and symptoms of depression plays a causal role in interpretation bias has yet to be examined.

Therefore, the main aim of the current study was to investigate the potential effect of ostracism on the interpretation of ambiguous information among individuals with depression symptoms. We hypothesized that ostracism, as opposed to other social experiences such as overinclusion, would lead to greater interpretation bias among individuals with high levels of depression symptoms. To examine the effect of social experience, we used a lab-controlled paradigm that manipulated ostracism and overinclusion (13). This paradigm enabled us to compare ostracism to another social experience in which the participant may feel conspicuous and self-aware *but not ignored or excluded* [i.e., overinclusion; (13)]. After undergoing the social manipulation, participants completed a computerized interpretation task that measured both direct (i.e., overt selection among two possible interpretations of an ambiguous sentence) and indirect [i.e., reaction time (RT)] selection of interpretation. Participants also reported their level of depression symptoms over the last week, thus enabling us to examine how the interaction between ostracism and depression symptoms affects interpretation bias.

## Method

### Participants

Seventy-one participants took part in the current study. One participant was removed from the analysis because of a lack of self-report measures due to technical problems. Power analysis using G^*^POWER software [version 3.1.9.7; (14)] confirmed that a sample size of 70 participants, alpha of 5% and medium effect size (*f*^2^ = 0.15), provided sufficient power (Power = 0.89) to conduct the study's analysis. Since ostracism has been found to affect men and women differently [e.g., (15)], only female participants took part in the study. Participants were students at the University of Haifa between the ages of 18 and 39 (*M* = 24.41, *SD* = 3.15). All were native speakers of Hebrew. Participants signed an informed consent form prior to participation and were debriefed at the end of the experiment. They received monetary compensation or course credit in exchange for their participation. They were randomly assigned by GraphPad software (16) to one of the two experimental social conditions: ostracism or overinclusion. The study was approved by the local ethics committee (approval no. 385/17).

### Materials

#### Social Experience (Ostracism/Overinclusion)

Social experience was manipulated using the Cyberball game, a computerized ball tossing game (13). The manipulation was conducted in line with the work of Zadro et al. (17), such that participants were misled to believe they were playing simultaneously with two other participants sitting in different rooms. In both conditions, the game lasted for 30 ball tosses. In the ostracism condition, participants obtained the ball only three times at the beginning of the game, while in the overinclusion condition they obtained the ball 15 randomly distributed times, more than any other “participant” in the game. We checked the manipulation in line with Zadro et al. (17): Participants reported the percent of throws they obtained in the game and used a 5-point scale to rate the level at which they believed they were included and/or ignored during the game.

#### Interpretation Bias

**Figure 1:**
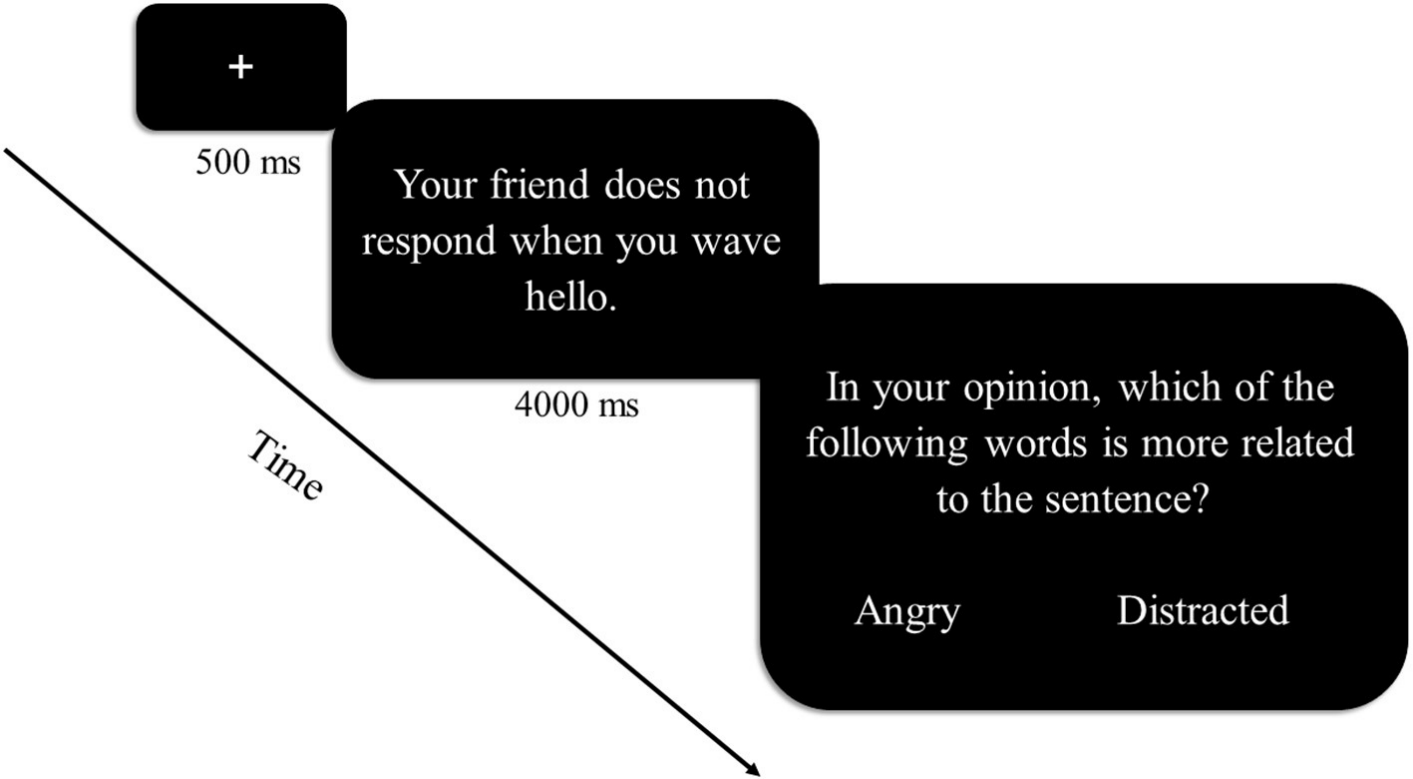
Sequence of a typical trial in the interpretation task. The sequence of a typical trial in the interpretation task is represented graphically. On each trial, a fixation point is followed by an ambiguous sentence describing a situation in which another person is involved. Participants are instructed to imagine themselves in the described situation. Each sentence is followed by negative and benign associative words that may be related to the sentence. Participants are asked to choose, as rapidly as possible, which word they believe is more related to the sentence.

Interpretation bias was measured by the interpretation task used by Richter et al. (18); for a graphical description of a typical trial in the task, see Figure 1. The task is a modification of the Word Sentence Association Paradigm [WSAP; (19, 20)], and was previously validated in Hebrew in subclinical (18) and clinical (21) samples of depression [for a detailed description of task validation please see (18)]. In this task, participants were shown 40 ambiguous sentences that appeared on the screen one at a time. Participants were instructed to try to imagine themselves in the described situations. All sentences described situations in which another person is involved. Each sentence was followed by presentation of negative and benign associative words related to the ambiguous sentence (e.g., “A friend has not returned your call” “busy/dodging”). Participants were asked to choose, as rapidly as possible, which word they believe is more related to the sentence. Four sentences were given at the beginning of the task as practice trials. For each participant, we calculated the percentage of selecting negative interpretations and the RT for selecting negative or benign interpretations. A *higher* percentage of negative interpretation selections, *lower* RTs for selecting *negative* interpretations and *higher* RTs for selecting *benign* interpretations are considered to be indicators of greater negative interpretation bias.

#### Depression Symptoms

Levels of depression symptoms were measured by the Depression and Anxiety Stress Scales [DASS-21; (22)]. The DASS-21 is a self-report questionnaire used to assess symptomatic levels of depression, anxiety and stress. The depression subscale consists of seven items, each rated on a 4-point scale. Participants reported their level of symptoms during the past week. Cronbach's alpha for internal consistency in the current sample was 0.85.

### Procedure

After signing a consent form, participants were told they were going to play an internet game known as Cyberball with two other participants playing in two different rooms. Participants then played the Cyberball game and subsequently completed the interpretation task. After that, participants completed the manipulation check and the DASS-21 questionnaire. To better characterize the sample, participants answered demographic questions, including country of origin, years of education, age, use of psychiatric medications and previous or current psychiatric diagnosis. The entire experimental procedure lasted about 30 min. At the end of the experiment, participants were debriefed and told that ostracism was part of the experiment (if they were in the ostracism condition).

### Data Analysis

#### Direct Measurement of Interpretation Bias (Model 1)

To examine the moderation effect of social experience (ostracism/overinclusion) on the association between levels of depression symptoms and interpretation bias, we conducted a two-step hierarchical regression on the direct measurement (i.e., selection of negative/benign interpretation). Levels of depression symptoms and social experience (ostracism/overinclusion) were entered into the regression as main effects in the first step, their interaction was entered in the second step, and the percentage of selecting negative interpretations was entered as the outcome.

#### Indirect Measurement of Interpretation Bias (Model 2–3)

Additional models focusing on indirect measures (i.e., selection RT) were further used to examine the moderation effect of social experience (ostracism/overinclusion) on the association between levels of depression symptoms and interpretation bias. Two models were conducted, one with mean RTs for selecting *negative* interpretations as an outcome (Model 2) and the other with mean RTs for selecting *benign* interpretations as an outcome (Model 3). Trials in which the RT measurement was above or below 2.5 standard deviations from the participant's mean RT were considered outliers and eliminated from the data. Nevertheless, even after cleaning RT outliers, fewer than 3% of each participant's total trials were eliminated.

Regression models were selected for the study's analysis in line with the statistical recommendations of Leppink (23).

## Results

### Manipulation Check

A series of *T*-tests indicated that the manipulation was effective. Participants in the ostracism condition reported feeling less included {*t*_(56_, _33)_ = 14.15, *p* = 0.001, Cohen's *d* = 6.22, 95% CI [3.01, 3.51]} and more ignored during the game {*t*_(61_, _42)_=25.99, *p* = 0.001, Cohen's *d* = 3.38, 95% CI [2.40, 3.19]} than participants in the overinclusion condition. Additionally, they reported obtaining fewer ball tosses during the game {*t*_(41, 98)_ = −10.88, *p* = 0.001, Cohen's *d* = 2.60, 95% CI [−38.78, −26.65]}.

### Descriptive Statistics and Pre-analysis Tests

Table 1 shows the descriptive statistics for symptoms of depression and direct and indirect interpretations in the two condition groups (ostracism/overinclusion). No between-group differences were observed in levels of depression symptoms [*t*_(68)_ = 1.10, *p* = 0.273].

**Table 1:** Descriptive statistics of depression symptoms and interpretation, by condition (ostracism/overinclusion).

	**Ostracism**			**Overinclusion**		
	**Mean**	**Std**	**Range**	**Mean**	**Std**	**Range**
Depression Symptoms	9.6	9.2	0.0–36.0	7.5	6.6	0.0–26.0
Percent of Selection of Negative Interpretation	30.6	14.6	08.0–60.0	28.9	12.9	0.0–63.0
RT for Selection of Negative Interpretation	2441.3	1047.1	1105.3–4934.5	1937.8	675.5	614.6–3811.1
RT for Selection of Benign Interpretation	1972.4	713.9	997.0–4401.1	1681.5	523.5	564.2–3042.3

*N = 70. RTs are shown in milliseconds*.

### Direct Measurement of Interpretation Bias

#### Model 1

The entire model was significant in predicting the percentage of selecting the negative interpretation [*F*_(3, 66)_= 12.61, *p* = 0.0001, Adjusted *R*^2^ = 0.33]. Levels of depression symptoms significantly and positively predicted the percentage of selecting the negative interpretation (β = 0.52, *p* = 0.000, 95% CI [0.04, 0.10]). Social experience (ostracism/overinclusion) by itself was not found to predict the percentage of selecting the negative interpretation (β = 0.06, *p* = 0.540, 95% CI [−0.02, 0.03]). In contrast, the social experience × depression symptoms interaction significantly predicted the percentage of negative interpretation selection (β =0 0.31, *p* = 0.003, 95% CI [0.01, 0.07], R^2^ change = 0.094).

**Figure 2:**
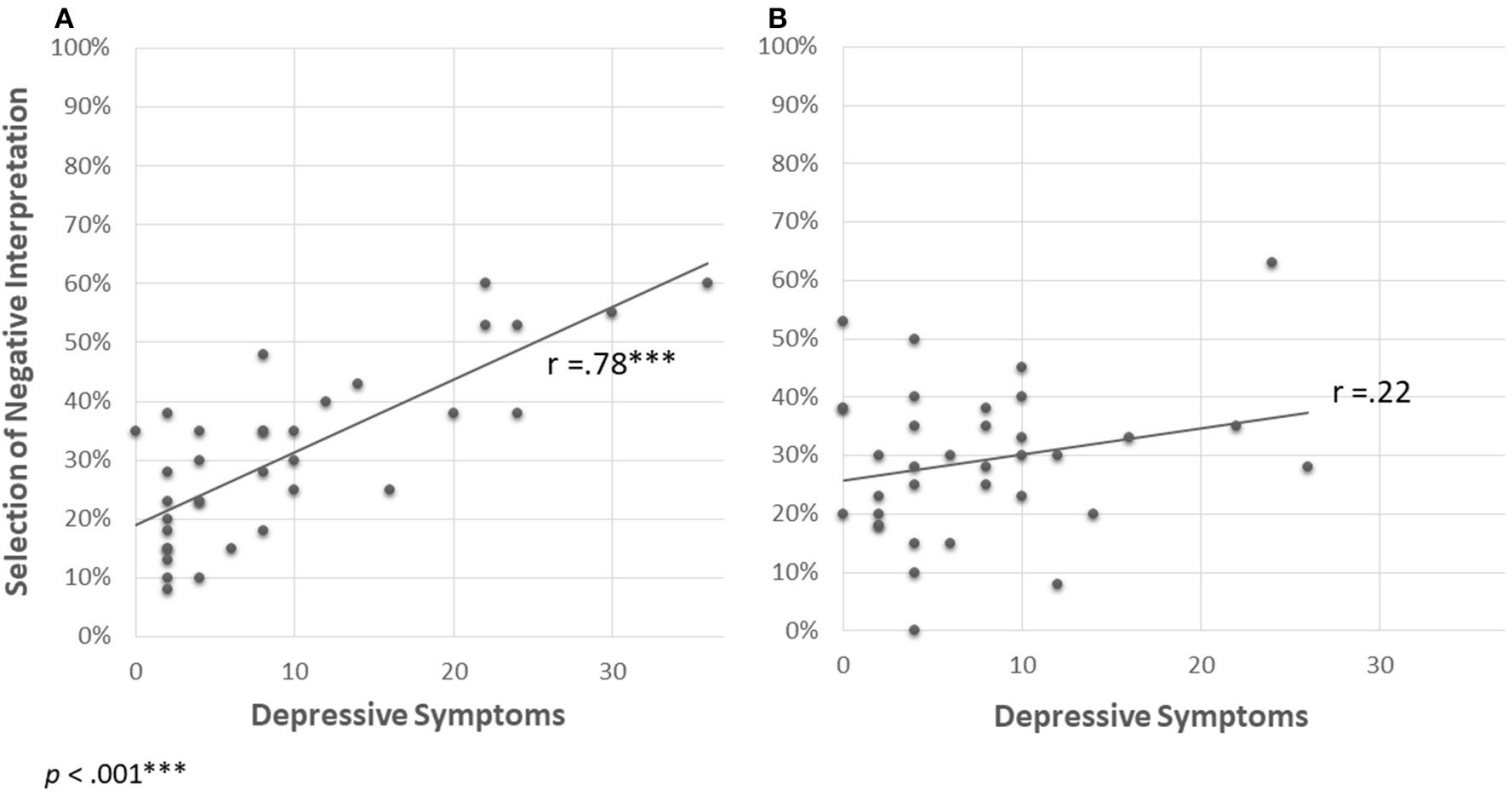
Prediction of selecting negative interpretation by levels of depression symptoms in each social experience condition [**(A)** ostracism, **(B)** overinclusion].

To better understand the interaction, we conducted two simple regressions that examined prediction of percentage of negative interpretation selection by levels of depression symptoms under the different social conditions (ostracism/overinclusion; see Figure 2). The regressions revealed that among individuals in the ostracism condition, levels of depression symptoms significantly and positively predicted percentage of negative interpretation selection (β = 0.78, *p* = 0.000, 95% CI [0.08, 0.1]; Figure 2A). In contrast, among individuals in the overinclusion condition, depression symptoms did not predict the percentage of negative interpretation selection (β = 0.22, *p* = 0.196, 95% CI [−0.02, 0.07]; Figure 2B). These results suggest that higher levels of depression symptoms predict higher levels of directly measured interpretation bias under conditions of ostracism but not under conditions of overinclusion.

### Indirect Measurement of Interpretation Bias

#### Model 2

The entire model was significant in predicting mean RTs for selecting a negative interpretation [*F*_(3, 65)_= 4.78, *p* = 0.005, Adjusted *R*^2^ = 0.14]. Levels of depression symptoms did not predict the mean RTs for selecting a negative interpretation (β = −0.08, *p* = 0.454, 95% CI [−282.57, 127.77]). In contrast, the social experience condition (ostracism/overinclusion) significantly and positively predicted mean RTs for selecting a negative interpretation (β = 0.28, *p* = 0.015, 95% CI [50.07, 456.63]): Participants in the ostracism condition selected a negative interpretation of ambiguous information faster than did participants in the overinclusion condition. Moreover, the social experience × depression symptoms interaction significantly predicted mean RTs for negative interpretation selection (β = −0.31, *p* = 0.007, 95% CI [−489.23, −78.89], R^2^ change = 0.096).

**Figure 3:**
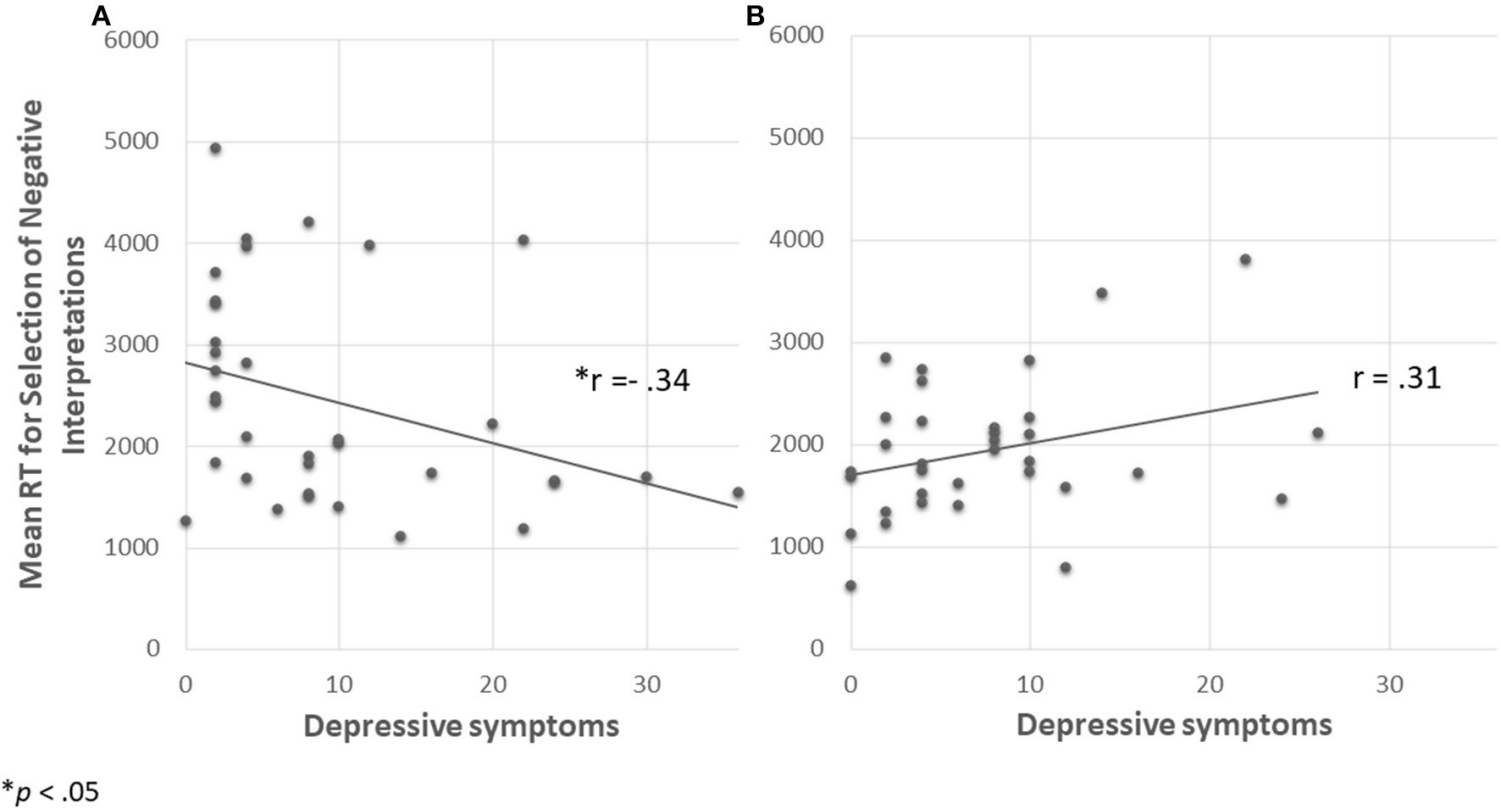
Mean RTs for selection of negative interpretation predicted by depression symptoms in each social experience condition [**(A)** ostracism, **(B)** overinclusion] Note that participants with higher depression symptoms selected negative interpretations faster in the ostracism than in the overinclusion condition.

To better understand the interaction, we conducted two simple regressions that examined prediction of mean RTs for selection of a negative interpretation by levels of depression symptoms under the different social conditions (ostracism/overinclusion; see Figure 3). The regressions revealed that among individuals in the ostracism condition, levels of depression symptoms significantly and positively predicted the mean RTs for selection of a negative interpretation (β = −0.34, *p* = 0.042, 95% CI [−709.49, −13.42]; Figure 3A). In contrast, among individuals in the overinclusion condition, depression symptoms did not predict the mean RTs for selection of a negative interpretation (β = 0.31, *p* = 0.07, 95% CI [−22.19, 435.51]; Figure 3B). These results suggest that higher levels of depression symptoms predict higher levels of interpretation bias, as measured by indirect measurements after experiencing ostracism but not after experiencing overinclusion.

#### Model 3

Levels of depression symptoms, social experience (ostracism/overinclusion) and their interaction were not found to be predictors of mean RTs for selecting benign interpretations [*F*_(3, 66)_ = 2.15, *p* = 0.102].

## Discussion

Given the role of interpretation bias in the etiology and maintenance of depression (2), it is especially important to understand *under which conditions* interpretation bias may emerge. The present findings suggest that social experiences contribute to interpretation bias. Specifically, ostracism—though not overinclusion—results in more and faster selection of *negative* interpretations of ambiguous social situations among individuals with higher levels of depression symptoms. Thus, when individuals with more intense symptoms of depression are socially ostracized, they are likely to interpret ambiguous situations in a negative manner more frequently and quickly than individuals who exhibit less intense symptoms of depression. These findings are consistent with theoretical knowledge, according to which social experiences may increase interpretation bias (5). These findings are also consistent with a previous study showing that greater sensitivity to ostracism is related to higher levels of depression symptoms and greater interpretation bias (24). Yet, whereas previous research contributed to understanding the aforementioned association, the present study expands previous knowledge by suggesting that ostracism enhances more negative interpretations of ambiguous information when levels of depression symptoms are higher. In line with Beck's theory of cognitive schemas (6), it is possible that among individuals with high levels of depression symptoms, social ostracism confirms latent negative cognitive schemas, which in turn increase their negative interpretations.

Replicating previous studies (4), the present findings suggest that levels of depression symptoms predict the selection of negative interpretations, such that higher levels of symptoms predicted a greater tendency to select negative interpretations. Yet whereas ostracism led to greater and faster selection of negative interpretations when levels of depression symptoms were higher, it did not affect RTs for the selection of *benign* interpretations. Additionally, levels of depression symptoms were not found to predict RTs for the selection of either negative or benign interpretations. This inconsistency between the direct and indirect measurements corresponds to the findings of previous studies that used indirect measurements of interpretation bias [i.e., (25)]. These studies showed interpretation bias in RTs for selection of negative but not of benign interpretations. This inconsistency is also in line with the findings of reviews [i.e., (26)] and meta-analyses [i.e., (4)] suggesting that the association between symptom levels and interpretation bias using direct measurements is consistent across studies, whereas the association between symptom levels and interpretation bias using indirect measurements yields mixed results.

The role of interpretation bias in the maintenance of depression symptoms has been emphasized and targeted across a variety of psychological treatments [i.e., Interpretation Bias Modification—(27); Cognitive Behavioral Therapy—(28); and psychodynamic therapy—(29)]. While these treatment orientations use different therapeutic interventions, they all invest major efforts in converting patients' maladaptive interpretations to more adaptive ones. By highlighting the effect of social ostracism on interpretation bias, the present findings add to accumulating knowledge regarding the possible factors by which interpretation bias maintains a depressive state [i.e., (30)]. Such knowledge may be utilized to treat individuals with depression symptoms by increasing the focus on maladaptive interpretations among patients reporting social ostracism.

The present findings also add to the accumulating literature on cognitive biases in psychiatric disorders and states, and particularly in depression [see (31), for elaboration]. Individuals with depression symptoms exhibit evidence of biased attention, interpretation, expectancy and memory (18). Beyond examining whether biased cognitions exist in psychiatric disorders, research has also placed emphasis on understanding the causal relations among cognitive biases [e.g., the effect of manipulated expectancies on participants' attention bias; (33)] and the relationships between the factors moderating cognitive biases [e.g., (32, 33)]. Studies also seek to use accumulating knowledge regarding cognitive biases to improve diagnosis of psychiatric disorders [e.g., see (18, 21) for an example of a diagnostic support system based on cognitive performance aimed at better differentiating between depression and anxiety diagnoses]. By shedding light on the interactive role of depression symptoms and aversive social experiences in the emergence of interpretation biases, the present study contributes to knowledge regarding the possible moderators of cognitive biases.

This study has several limitations. First, the examination was restricted to women, leaving open the question of whether and how the combined effect of depression symptoms and social experience (ostracism/overinclusion) may affect interpretation bias among men. The present study also examined levels of depression symptoms as distributed in the general population, whereas the findings may be different in a clinical sample. In addition, the present study used overinclusion as a control condition and not the more common inclusion condition (33% ball tosses for each “participant”). It is possible that the study's results would be different when using other control conditions. Additionally, overinclusion may not necessarily be a positive experience for everyone (34). Therefore, future studies should examine the combined effect of depression symptoms and social experience on interpretation bias among both men and women, while also considering clinical levels of depression symptoms and controlling for complicated feelings that may raise under conditions of overinclusion. Furthermore, the study's design does not allow us to infer whether the interaction between depression symptoms and ostracism directly affects interpretation bias or whether this effect is mediated by other factors, such as negative feelings or schemas elicited by ostracism. Future studies should further examine this issue.

The present findings highlight the importance of examining how social experiences such as ostracism interact with depression symptoms in order to understand the conditions under which interpretation bias may emerge. By doing so, the current research broadens our understanding regarding the role of interpretation bias in the maintenance of depression symptoms. Such an understanding may be utilized in the future to develop treatments tailored to each individual's cognitive biases and personal experiences.

## Data Availability Statement

The raw data supporting the conclusions of this article will be made available by the authors, without undue reservation.

## Ethics Statement

The studies involving human participants were reviewed and approved by Ethics Committee, School of Psychological Sciences, University of Haifa, Israel. The patients/participants provided their written informed consent to participate in this study.

## Author Contributions

AB-S ran the research, analyzed the data, and wrote the first draft of the manuscript. HO-S initiated and supervised the study. TR participated in the design, data analysis, and writing. SZ-M advised in the process of designing and implementing the study. All authors participated in the design and planning of the study and approved the final manuscript.

## Funding

This research was supported by a grant from the Ministry of Science & Technology, Israel, Ministry of Europe and Foreign Affairs (MEAE) and the Ministry of Higher Education, Research and Innovation (MESRI) of France awarded to HO-S.

## Conflict of Interest

The authors declare that the research was conducted in the absence of any commercial or financial relationships that could be construed as a potential conflict of interest.

## Publisher's Note

All claims expressed in this article are solely those of the authors and do not necessarily represent those of their affiliated organizations, or those of the publisher, the editors and the reviewers. Any product that may be evaluated in this article, or claim that may be made by its manufacturer, is not guaranteed or endorsed by the publisher.
